# Protective Effect of the Polyphenol Ligustroside on Colitis Induced with Dextran Sulfate Sodium in Mice

**DOI:** 10.3390/nu16040522

**Published:** 2024-02-13

**Authors:** Ruonan Gao, Yilin Ren, Peng Xue, Yingyue Sheng, Qin Yang, Yuanyuan Dai, Xiaoyue Zhang, Ziming Lin, Tianhao Liu, Yan Geng, Yuzheng Xue

**Affiliations:** 1 Department of Gastroenterology, Affiliated Hospital of Jiangnan University, Wuxi 214122, China; 2 Wuxi School of Medicine, Jiangnan University, Wuxi 214122, China; 3 Key Laboratory of Industrial Biotechnology of Ministry of Education, School of Biotechnology, Jiangnan University, Wuxi 214122, China; 4 School of Medicine, Nantong University, Nantong 226001, China; 5 School of Life Science and Health Engineering, Jiangnan University, Wuxi 214122, China; gengyan@jiangnan.edu.cn

**Keywords:** inflammatory bowel disease (IBD), ligustroside, colitis

## Abstract

Dietary polyphenols are reported to alleviate colitis by interacting with gut microbiota which plays an important role in maintaining the integrity of the intestinal barrier. As a type of dietary polyphenol, whether ligustroside (Lig) could alleviate colitis has not been explored yet. Here, we aimed to determine if supplementation of ligustroside could improve colitis. We explored the influence of ligustroside intake with different dosages on colitis induced with dextran sulfate sodium (DSS). Compared to the DSS group, supplementation of ligustroside could reduce body weight (BW) loss, decrease disease activity indices (DAI), and relieve colon damage in colitis mice. Furthermore, ligustroside intake with 2 mg/kg could decrease proinflammatory cytokine concentrations in serum and increase immunoglobulin content and antioxidant enzymes in colon tissue. In addition, supplementation of ligustroside (2 mg/kg) could reduce mucus secretion and prevent cell apoptosis. Also, changes were revealed in the bacterial community composition, microbiota functional profiles, and intestinal metabolite composition following ligustroside supplementation with 2 mg/kg using 16S rRNA sequencing and non-targeted lipidomics analysis. In conclusion, the results showed that ligustroside was very effective in preventing colitis through reduction in inflammation and the enhancement of the intestinal barrier. Furthermore, supplementation with ligustroside altered the gut microbiota and lipid composition of colitis mice.

## 1. Introduction

Inflammatory bowel disease (IBD) is a chronic non-specific intestinal inflammatory disease. It mainly involves colonic mucosa and submucosa and presents as recurrent chronic intestinal inflammation. There are two subtypes of IBD, including ulcerative colitis (UC) and Crohn’s disease (CD) [1,2]. The latest data show that IBD is still on the rise worldwide, with about 0.2% of the European population suffering from IBD, which has become a serious global health burden [3]. The pathogenesis of IBD is unknown at present, involving a variety of causes such as being heredity, the environmental diet, intestinal barrier, immunity, and microorganisms [4,5,6]. IBD is prone to recurrence and various systemic complications after treatment, which seriously affect patient’s quality of life and survival rate. How to effectively solve the treatment difficulties of IBD has become a major research focus at present.

Several factors contribute to IBD pathogenesis, including genetic susceptibility and environmental factors, which can weaken the intestinal barrier and lead to inappropriate intestinal immune activation by affecting the microflora. An imbalance of intestinal microflora has been observed in patients with IBD [7]. The most common changes were that Firmicutes decreased and Enterobacteriaceae increased in IBD [8,9]. Compared with healthy individuals, *Faecalibacterium prausnitzii*, *Roseburia intestinalis*, Lachnospiraceae, and Ruminococcaceae decreased, while *B.fragilis*, *Escherichia coli*, and Enterobacteriaceae increased in CD. *Bifidobacterium*, *Roseburia hominis*, and *Faecalibacterium prausnitzii* decreased while Lachnospiraceae increased in UC [10,11]. In addition, an imbalance between intestinal mucosa and intestinal contents is also a cause of pathological changes in IBD. Intestinal mucosa consists of epithelial cells, goblet and Paneth cells, stroma, and immune cells. Goblet cells can produce a mucous matrix that covers epithelial cells and thus plays a role in mucosal defense and repair [12]. Innate immune cells, such as neutrophils and macrophages, can strengthen the physical and functional barriers of the intestinal barrier as the first line of defense of the well-developed mucosal innate immune system. Neutrophils can directly cause tissue damage by releasing neutrophil elastase, matrix metalloproteinases (MMPs), pro-inflammatory cytokines including tumor necrosis factor (TNF)-α and interleukin (IL)-1β, and superoxide dismutase (SOD) in IBD. Furthermore, these factors lead to damage to the epithelial barrier as well as recruiting neutrophils and other immune cells to the inflamed area [13,14]. In addition, macrophages were expressed at high levels of pro-inflammatory molecules including TNF-α, IL-1β, IL-6, and inducible nitric oxide synthase (iNOS) during IBD [15].

Polyphenols are organic compounds containing oxygen heterocycles. Researchers have found that polyphenols and their derivatives can play antioxidant and anti-inflammatory roles by regulating intestinal barrier function, changing the composition of intestinal flora, or activating congenital and adaptive immune responses [16,17,18]. On the one hand, colonic microbes extensively metabolize polyphenols [19]. Polyphenols can shape the composition of the intestinal bacteria, such as through increasing probiotics *Bifidobacterium* and inhibiting the growth of several pathogenic bacteria [20]. On the other hand, it has been demonstrated that polyphenols have protective effects on intestinal barrier functions by modulating mucus production and antimicrobial peptide secretion [21,22]. As a functional food, ligustroside is one of the common polyphenolic compounds which can be extracted from extra virgin olive oil (EVOO) [17,23]. Diets including EVOO in mice colitis have been extensively studied [24,25]. The prospect of ligustroside being a complementary therapy for IBD has attracted more attention in recent years. Therefore, considering that the effects of ligustroside on colitis are unclear, we investigated the potential effects of ligustroside on DSS-induced colitis in mice.

## 2. Materials and Methods

### 2.1. Animal Experimental Design

**Figure 1:**
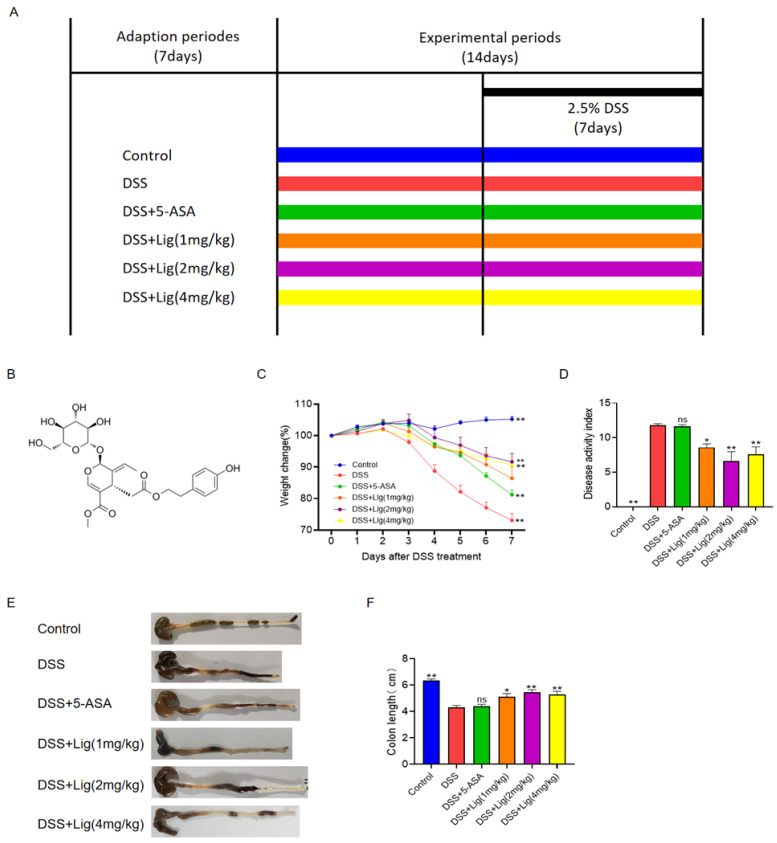
Supplementation of ligustroside alleviated colitis symptoms in DSS-treated mice. (**A**) Treatment timeline for the experiment, (**B**) chemical structure of ligustroside, (**C**) body weight change (%), (**D**) disease activity index scores, (**E**,**F**) colon lengths of the mice. * *p* < 0.05 and ** *p* < 0.01 vs. DSS group, ns means *p* > 0.05 vs. DSS group, *n* = 5.

Male C57BL/6 mice (aged 7–8 weeks, weighing about 20 ± 2 g) were purchased from Gempharmatech Co., Ltd. (Nanjing, China) The drug ligustroside (CAS35897-92-8, purity ≥ 98%, store at 2–8 °C) and 5-aminosalicylic acid (5-ASA, CAS89-57-6, purity ≥ 99%, store at RT) were purchased from Shanghai Yuanye Bio-Technology Co., Ltd. (Shanghai, China), and dextran sulfate sodium (DSS, CAS9011-18-1, store at 2–8 °C, M.W 40,000) was purchased from Shanghai Macklin Biochemical Co., Ltd. (Shanghai, China). As shown in Figure 1A, a total of 30 C57BL/6 male mice were randomly divided into six groups (*n* = 5 per group).

After 7 days of adaption, mice in the Control and DSS groups received phosphate-buffered saline (PBS) solution while mice in the DSS + 5-ASA group received oral administration of 5-ASA dissolved in distilled water (150 mg/kg BW) and mice in the DSS + Lig groups received different dosages of ligustroside dissolved in distilled water daily for 14 days. On Day 8, 2.5% DSS (*w*/*v*) was added to the water for mice except for the Control group for an additional 7 days. All mice were raised in the Experimental Animal Centre of the Medical College of Jiangnan University. Mice were fed a rodent chow diet, and the daily food intake of the mice was about 5–10 g/mouse. The mice ate and drank freely every day. The feeding conditions were as follows: 12 h light/dark cycle, temperature 22 ± 2 °C, humidity 55 ± 5%, and noise level ≤ 60 dB. All mice were anesthetized through inhalation of 3% isoflurane and then euthanatized using cervical dislocation on the 15th day of the experiment. A review and approval of the animal study was conducted by Jiangnan University’s Institutional Animal Care and Use Committee [Approval No. 20220330c1440615 (114)].

### 2.2. Disease Activity Index (DAI)

The disease changes in mice were observed by evaluating their DAI, including weight loss, occult blood or blood stool and shape of feces, as described in previous research article [26].

### 2.3. Analysis of Colon Histology in Mice

After the experiment, mice’s colons were dissected. The lengths of the colons were measured. Ice-cold phosphate-buffered saline was used to wash the lumen of the colons. A neutral tissue fixation solution was then applied to the tissues. After dehydration with ethanol, paraffin was used to embed the sample. After preparing 4 mm thick paraffin sections, hematoxylin and eosin staining, and Alcian blue/periodic acid-Schiff (AB/PAS) staining were conducted. Under the microscope, colon morphology was observed. As previously described, the degree of tissue injury was based on the degree of inflammatory cell infiltration (0–3) and tissue injury (0–3) [27]. A TUNEL assay was used to determine the apoptosis level in colonic cells. The nuclei of the cells were stained with DAPI and observed using a fluorescence microscope.

### 2.4. Enzyme-Linked Immunosorbent Assay (ELISA)

We separated mouse serum from blood with centrifugation at 1500× *g* for 15 min and stored at −20 °C. The contents of proinflammatory cytokines IL-6, IL-1β, TNF-α, and immunoglobulin IgA, IgG, and IgM in serum were detected using an ELISA kit (Thermo Scientific, Shanghai, China). The colonic tissue of mice was homogenized in ice-cold phosphate-buffered saline and centrifuged at 13,000× *g* for 20 min at 4 °C. The supernatant of colonic tissue was collected, and the content of the secretory immunoglobulin (SIgA) and superoxide dismutase (SOD) in the colonic tissue supernatant was detected using an ELISA kit.

### 2.5. Gut Microbiota Analysis

DNA from the stool microbial community was extracted using MagPure Stool DNA KF kit B (Magen, Foshan, China), and then it was quantified with a Qubit Fluorometer using the Qubit dsDNA BR Assay kit (Invitrogen, Waltham, MA, USA). The PCR products were sequenced on the Illumina MiSeq platform (BGI, Shenzhen, China). Tags with 100% similarity were clustered to the same ASV. The Ribosomal Database Project Bayesian classifier algorithm was used to analyze the ASV representative sequences. Alpha and beta diversity analyses were assessed using MOTHUR and QIIME (v2022.2). Linear Discriminant Analysis Effect Size (LEfSe) was used to analyze the biomarkers of different groups. Microbial functions were also predicted using Phylogenetic Investigation of Communities through Reconstruction of Unobserved States (PICRUSt).

### 2.6. Non-Targeted Lipidomic Analysis

To extract metabolites from the contents of the mouse colons, 300 μL methanol, 1000 μL methyl tert-butyl ether, and 250 μL ultra-pure water extraction solvent was added to the sample and thoroughly vortexed. Then, the samples were incubated for 10 min, followed by centrifugation at 1000× *g* for 5 min at 10 °C. We collected the supernatant, and a vacuum centrifuge was used to collect and dry the elution. For a liquid chromatography mass spectrometer (LC-MS) analysis, the samples were redissolved in 50 μL chloroform/methanol (*v*:*v* = 2:1) solvent and transferred to vials.

An ultra-high-performance liquid chromatography (UHPLC) system (ThermoFisherQ Exactive™ Plus, Waltham, MA, USA) was used to analyze the samples. The sample was separated using a UPLC BEH C18 column (100 mm × 2.1 mm, 1.7 μm). Separation was initiated at a flow rate of 250 μL/min. During the whole analysis process, the sample was kept at 4 °C. Detection of metabolites was performed using ThermoFisherQ Exactive™ Plus with an ESI ion source. Raw data were processed using LipidSearch v.4.1 (Thermo Fisher Scientific, Waltham, MA, USA). The data were imported into MetaboAnalysit 5.0 for sparse PLS-DA (sPLSDA), volcano map, and hierarchical clustering heatmap analysis.

### 2.7. Statistical Analyses

The data are shown as means ± standard error of the mean (SEM). The statistical difference between two groups was assessed using unpaired two-tailed Student’s *t*-tests, and the differences of more than two groups were assessed using ordinary one-way analysis of variance, using GraphPad Prism 8.4 (Graphpad Software, Inc., La Jolla, CA, USA). A difference of *p* < 0.05 was considered statistically significant. * *p* < 0.05 and ** *p* < 0.01 vs. DSS group, ns means *p* > 0.05 vs. DSS group, *n* = 5.

## 3. Results

### 3.1. Ligustroside Can Relieve DSS-Induced Colitis in Mice

The chemical structure of ligustroside is shown in Figure 1B. The timeline of the experiment is shown in Figure 1A. After drinking 2.5% DSS solution for one week, mice developed severe acute colitis, including loss of weight (Figure 1C), an increase in the DAI (Figure 1D), diarrhea, and bleeding in feces. DSS stimulation significantly shortened the length of the colon in mice (Figure 1E,F). After the administration of DSS solution for one week, the weight of the mice significantly dropped, which was alleviated through the oral administration of ligustroside (Figure 1C). In colitis mice induced with DSS, ligustroside also significantly decreased the DAI scores (Figure 1D). Ligustroside increased the length of the colon (Figure 1E,F). To summarize, DSS-induced colitis could be significantly improved with oral ligustroside supplementation.

### 3.2. Ligustroside Can Ameliorate Colonic Injury Induced with DSS

**Figure 2:**
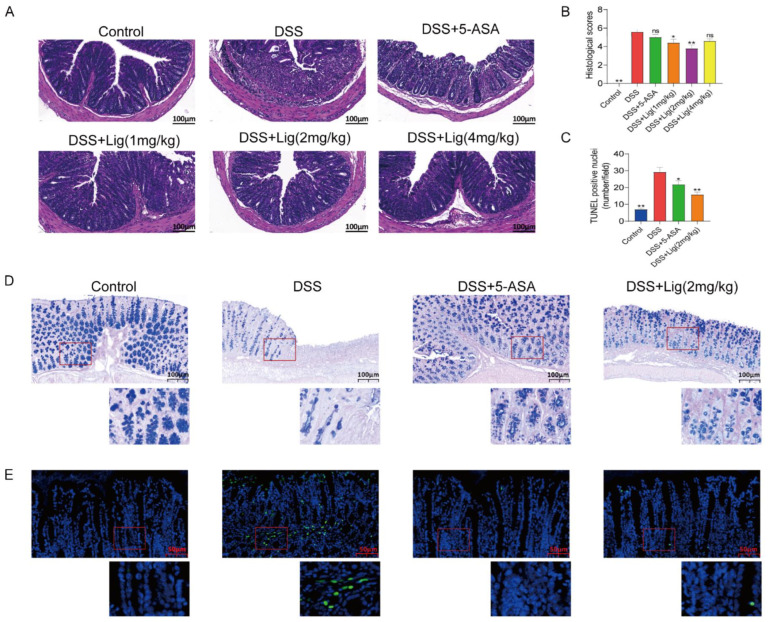
Supplementation of ligustroside alleviated histological changes in colitis mice. (**A**) Mouse colon morphology stained with hematoxylin and eosin. (**B**) The histological scores of mouse colon tissue. (**C**) The number of TUNEL-positive nuclei per field in mouse colon. (**D**) AB/PAS staining. (**E**) The TUNEL staining of colon cells. * *p* < 0.05 and ** *p* < 0.01 vs. DSS group, ns means *p* > 0.05 vs. DSS group, *n* = 5.

A histopathological analysis showed that mucosal injury, inflammatory cell infiltration, and crypt loss were significantly alleviated in the mice treated with ligustroside (Figure 2A), while the histopathological scores of colons from the DSS group were significantly higher than the Control group. A significant decrease in the histopathological scores of the colon tissue was observed in mice after supplementation with ligustroside (Figure 2B).

Based on the results of the above data, we found that the DSS + Lig (2 mg/kg) group had the best effect on alleviating colitis induced with DSS. Therefore, we conducted further analysis on the mice in the DSS + Lig (2 mg/kg) group. AB-PAS staining analysis showed that the protective substances secreted by the mucus layer in the colon of mice decreased in the DSS group (Figure 2D), and the intestinal barrier was damaged. Furthermore, a large number of apoptotic cells were seen in the DSS group using TUNEL staining (Figure 2C,F), which could be reversed through the supplementation of ligustroside.

### 3.3. Effects of Ligustroside on the Secretion of Cytokines, Immunoglobulins, and the Oxidation Index

**Figure 3:**
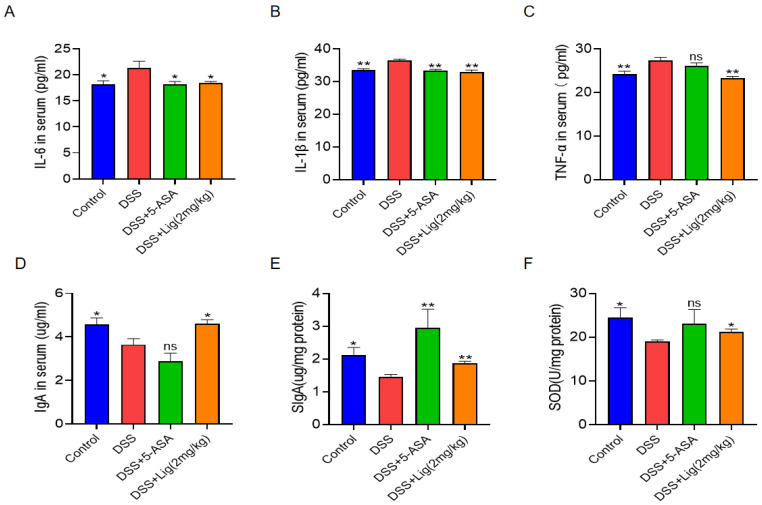
(**A**–**D**) Concentrations of IL-6, IL-1β, TNF-α, and IgA in the serum of colitis mice. (**E**,**F**) Concentrations of SIgA and SOD in colon tissue of colitis mice. * *p* < 0.05 and ** *p* < 0.01 vs. DSS group, ns means *p* > 0.05 vs. DSS group, *n* = 5.

The concentrations of proinflammatory cytokines IL-6, IL-1β, and TNF-α significantly increased in the serum of mice with colitis induced with DSS (Figure 3A–C), while the concentration of immunoglobulin IgA in serum was decreased in the DSS group (Figure 3D). Furthermore, the concentration of SIgA (Figure 3E) and the activity of the antioxidant SOD (Figure 3F) in the mouse colon were decreased in the DSS-induced colitis mice. However, after supplementing with ligustroside, the changes in these indicators can be reversed and were similar to the Control group.

### 3.4. Gut Microbiota Profiling

**Figure 4:**
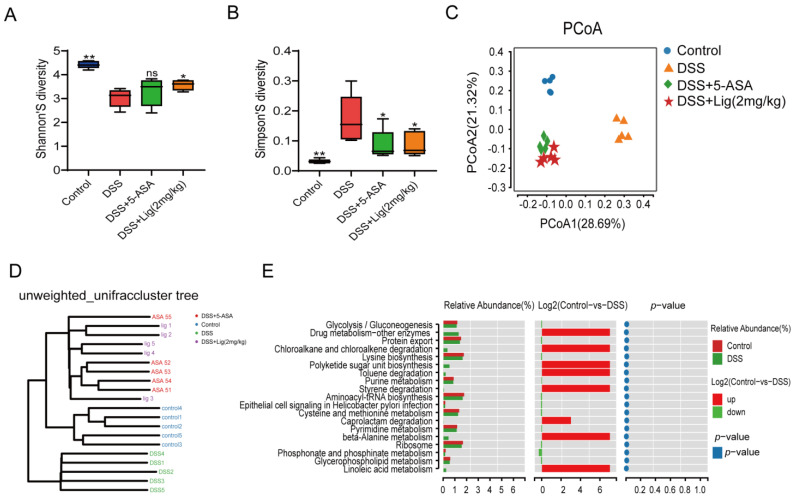
The microbiota of mice suffering from colitis were altered by supplementation with ligustroside. (**A**,**B**) Shannon and Simpson indices showed alpha diversity, (**C**) principal co-ordinate analysis of gut microbiota, (**D**) unweighted_UniFrac cluster tree, (**E**) functional difference analysis between Control and DSS groups. * *p* < 0.05 and ** *p* < 0.01 vs. DSS group, ns means *p* > 0.05 vs. DSS group, *n* = 5.

In total, 16s rRNA was sequenced from the feces of mice. Alpha diversity was shown using the Shannon and Simpson indices. We found that the Shannon index decreased significantly (Figure 4A), while the Simpson index increased (Figure 4B) after treatment with DSS. Principal co-ordinate analysis showed that the intestinal microflora of mice changed significantly after treatment with DSS. The intestinal microbial composition of the mice treated with ligustroside was more similar to that of the 5-ASA group (Figure 4C). At the same time, an unweighted_UniFrac cluster tree analysis showed that the microbial composition of the Control group, DSS + 5-ASA group, and DSS + Lig (2 mg/kg) group were more similar (Figure 4D). Using functional difference analysis, it was found that there were significant differences in glycerophospholipid metabolism and linoleic acid metabolism pathways between the Control and DSS groups (Figure 4E). In view of this change, we decided to explore the effect of ligustroside intervention on lipid metabolism in colitis mice.

**Figure 5:**
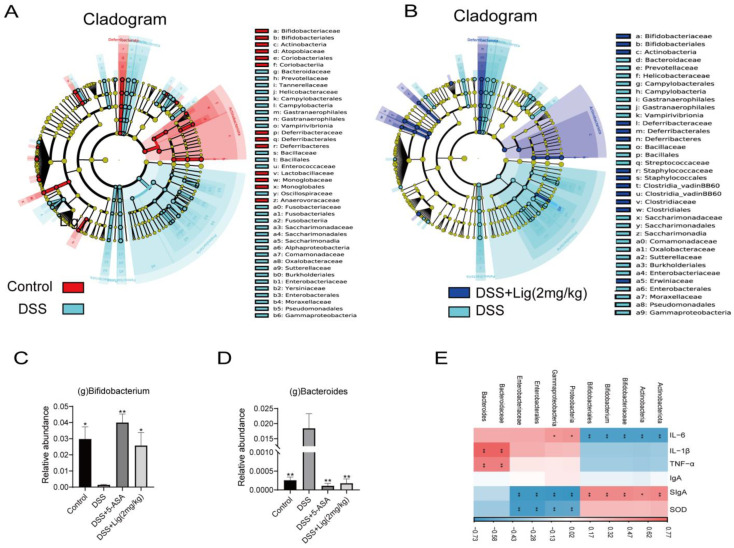
(**A**) Taxonomic cladogram of LEfSe analysis between Control and DSS groups. Biomarker taxa are shown in different colors, where Linear discriminant analysis (LDA) score > 2 and *p* < 0.05, (**B**) taxonomic cladogram of LEfSe analysis between the DSS + Lig (2 mg/kg) and DSS groups. Biomarker taxa are shown in different colors, where LDA score > 2 and *p* < 0.05. Relative abundance of Bifidobacterium (**C**) and Bacteroides (**D**) are shown. (**E**) Correlation between different key bacteria and inflammation-related indices. * *p* < 0.05 and ** *p* < 0.01 vs. DSS group, ns means *p* > 0.05 vs. DSS group, *n* = 5.

Then, LEfSe analysis was used to analyze the differences between groups. It was found that Actinobacteriota, Actinobacteria, Bifidobacteriales, Bifidobacteriaceae, and *Bifidobacterium* were the key differential species in the Control group, but Proteobacteria, Gammaproteobacteria, Enterobacterales, Enterobacteriaceae, Bacteriodaceae, and *Bacteroides* are the key differential species in the DSS group (Figure 5A, Supplementary Figure S2A). The LEfSe analysis of the DSS + Lig (2 mg/kg) group and DSS group showed that the key differential species in the DSS + Lig (2 mg/kg) group were similar to those in the Control group, in which the main differential bacteria were *Bifidobacterium*, while *Bacteroides* was still the key differential species in the DSS group (Figure 5B, Supplementary Figure S2B). In addition, these key different species also had significant differences among the four groups, and they were statistically significant (Figure 5C,D, Supplementary Figure S3A–I). Then, by analyzing the correlation between different key bacteria and inflammation-related indexes (Figure 5E), it was found that *Bifidobacterium* had a positive correlation with index SIgA and SOD after the intervention of ligustroside, and had a significant positive correlation with SIgA. In addition, it was negatively correlated with IL-6, IL-1β, and TNF-α and significantly negatively correlated with IL-6. On the contrary, *Bacteroides* showed the opposite trend. Based on the above results, we speculated that ligustroside could reshape the gut microbiota and restore a damaged intestinal barrier. Furthermore, ligustroside can increase the proportion of beneficial bacteria *Bifidobacterium* in the intestine that have anti-inflammatory effects. In addition, the improvement of the intestinal microenvironment can also reduce abnormal activation of innate immunity, prevent abnormal activation and aggregation of neutrophils, and thus reduce the production of pro-inflammatory cytokines such as IL-6 to alleviate damage to the intestinal barrier.

### 3.5. Non-Targeted Lipidomic Analysis

**Figure 6:**
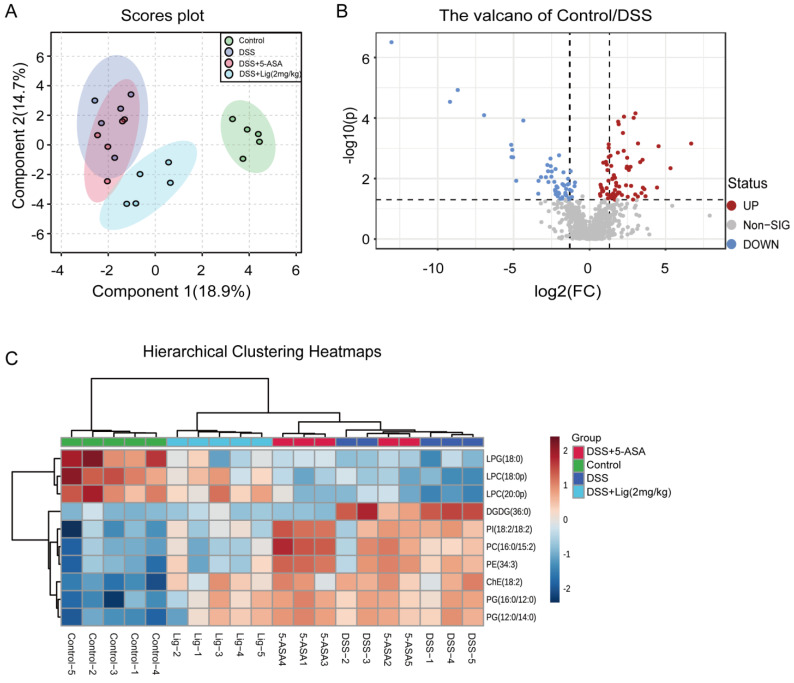
(**A**) Score plots for the sPLSDA model of metabolites in the intestinal content of mice, (**B**) volcano plot of significantly different metabolites between the Control and DSS groups (fold change > 1.5 and *p* < 0.05), (**C**) hierarchical clustering heatmaps of differential metabolites in intestinal luminal contents of mice between different groups. The red color indicates a metabolite concentration higher than the mean concentration of all samples, and the blue color indicates a metabolite concentration lower than the mean concentration of all samples, *n* = 5.

Using UHPLC-QE-MS to analyze the colonic contents of mice, a total of 914 peaks were obtained in positive and negative ion mode. sPLSDA found a significant difference between the composition of metabolites in the DSS + Lig (2 mg/kg) group and the DSS group (Figure 6A). Furthermore, the lipidomic metabolites in the DSS + Lig (2 mg/kg) group were nearer to the Control group than the DSS group. Then, we detected the differential metabolites between the Control and DSS groups using a volcano map. We found that there were 130 key different metabolites between the Control and DSS groups. Among these metabolites, 57 metabolites were significantly decreased and 73 metabolites were significantly up-regulated (Figure 6B). In addition, among the top 10 metabolites with the most obvious changes in Hierarchical Clustering Heatmaps (Figure 6C), Lyso-phosphatidylglycerol (LPG) (18:0), Lyso-phosphatidylcholine (LPC) (18:0p), and LPC (20:0p) decreased significantly in the DSS group. The other seven substances, Digalactosyldiacylglycerol (DGDG) (36:0), Phosphatidylinositol (PI) (18:2/18:2), Phosphatidylcholine (PC) (16:0/15:2), Phosphatidylethanolamine (PE) (34:3), Cholesteryl Ester (ChE) (18:2), Phosphatidylglycerol (PG) (16:0/12:0), and PG (12:0/14:0) significantly increased in the DSS group.

## 4. Discussion

In this study, the polyphenolic compound ligustroside was used as an intervention for the first time, to the best of our knowledge, to explore its supplementary therapeutic effects on DSS-induced colitis in mice.

IBD, as chronic intestinal inflammation, has become an important worldwide public health problem [28]. At present, IBD sufferers are still prone to relapse after treatment, and the course of the disease is protracted, seriously affecting the quality of life of patients. Therefore, we have attempted to find new compounds, derived from food or other substances, that can be used as supplementary treatment to improve the treatment of IBD. Several studies have shown that among people who suffer from certain chronic diseases associated with oxidative stress, inflammation, and the immune system, the Mediterranean diet, which contains ligustroside, may have protective effects [23].

DSS has been widely used to induce colitis and IBD in mice [29]. The mice utilized in this study developed acute colitis after drinking a solution of 2.5% DSS, resulting in diarrhea, hematochezia, weight loss, and colon shortening. 5-ASA is a classic drug used for the treatment of IBD, and it has been used in many studies to treat acute colitis induced with DSS [30,31]. In this study, it was found that supplementation of 5-ASA and ligustroside could significantly improve intestinal inflammation in mice with colitis, increase BW and colon length along with a reduction in DAI scores.

Many studies have shown that cytokines IL-6, IL-1β, and TNF-α play an important role in colitis [28]. Their increased expression can lead to intestinal dysfunction [32]. Oxidative stress is also involved in the pathogenesis and aggravation of IBD [33]. In this study, mice with colitis had more serious colonic mucosal damage than mice in the Control group, as well as an increased number of inflammatory cells. Significantly higher concentrations of proinflammatory cytokines were found in the serum of colitis mice than in the serum from Control group mice, and these inflammatory symptoms could be alleviated after intervention with ligustroside. The change of SOD content is an important index of oxidative stress in vivo, and DSS treatment can reduce the activity of SOD [34]. The intestine is rich in a large amount of SIgA, which is very important for mucosal immunity. It protects the mesentery and has therapeutic effects against IBD [20]. The content of SIgA in the intestinal tissue and immunoglobulin IgA in the serum of colitis mice decreased, but recovered after the intervention with ligustroside.

In DSS-induced colitis mice, DSS can directly target intestinal mucosa, destroy intestinal epithelial cells of the basal recess, damage the integrity of the mucosal barrier, and significantly change the composition of intestinal microorganisms [35]. Therefore, we studied the effect of ligustroside on the histopathological changes in the colon and on intestinal microfloral imbalance after DSS intervention. Our results showed that the intestinal integrity and intestinal microfloral composition in the DSS-treated mice were improved with supplementation of ligustroside. Compared with the Control group, supplementation of ligustroside changed the composition of lipid metabolites in the intestinal contents of the mice. In this study, we also found that the total number of microbial species in colitis mice decreased compared with the Control group. In the LEfSe analysis between Control and DSS groups, we found that *Bifidobacterium*, one of the bacteria beneficial to colitis [36], became the key differential microorganism in the Control group, and the proportion of *Bifidobacterium* was different between the Control and DSS groups. In contrast, in the DSS group, *Bacteroides* became the key differential genus, and the genus level was higher than that of the Control group, a finding which is similar to the previous research [37]. According to our findings, supplementation of ligustroside can improve species composition and species diversity in the gut of DSS-induced colitis mice.

As previous studies have shown, the lipid metabolism profiles of patients with IBD change [38]. Chronic inflammation on the surface of the intestinal epithelium has been found to be regulated by lipids in IBD [39]. Compared with healthy mice, the lipid metabolism spectrum of colitis mice induced with DSS also changed significantly. In addition, as important structural components of cell membranes, lipids have significant effects on different metabolic pathways and cell functions, which are important components of the intestinal barrier. In our results, a significant difference was found in the composition of colonic contents between the Lig and DSS groups. The composition of colonic contents in the Lig group was more similar to that of the Control group than the DSS group. Based on these findings, it is clear that DSS treatment can cause disruption of the lipid metabolism by altering the colonic lipid profile, and ligustroside protected it by reversing this process. Furthermore, endogenous bioactive lipids are widely recognized as pro-inflammatory mediators and triggers of inflammatory bowel disease [40,41]. These results revealed a close relationship between lipid metabolism and inflammation, suggesting that exploring lipid metabolism further may provide a way to regulate IBD’s inflammatory response.

## 5. Conclusions

Under the existing treatment methods for IBD, ligustroside may be useful as a supplementary treatment to improve the treatment effect and long-term quality of life for IBD patients. In conclusion, supplementation of ligustroside could improve the occurrence of colitis in mice by reducing inflammation, enhancing the intestinal barrier, improving intestinal microbial composition, and altering lipid metabolism composition. However, it is not without its limitations: results from animals need to be extrapolated to humans, potential gaps need to be filled, other IBD models need to be validated, and so forth. These issues deserve further study and in-depth analyses from researchers.

## Data Availability

Data are contained within the article and Supplementary Materials.

## Supplementary Materials

The following supporting information can be downloaded at: https://www.mdpi.com/article/10.3390/nu16040522/s1, Figure S1. The concentrations of IgG (A) and IgM (B) in serum. Data were presented as means ± SEM. * *p* < 0.05 and ** *p* < 0.01 vs. DSS group, ns means *p* > 0.05 vs. DSS group, *n* = 5. Figure S2. Top ten Linear discriminant analysis (LDA) score for different taxa abundances (A) between control and DSS groups and (B) between DSS + Lig (2 mg/kg) and DSS groups, *n* = 5. Figure S3. Relative abundance of predominant bacteria is shown for the phylum (A,B), class (C,D), order (E,F) and family (G–I) levels. Data are presented as means ± SEM. * *p* < 0.05 and ** *p* < 0.01 vs. DSS group, ns means *p* > 0.05 vs. DSS group, *n* = 5. Figure S4. (A) The quantification of AB/PAS Positive cells/crypt in colon tissue. (B) Mucus thickness of colon tissue. Data are presented as means ± SEM. * *p* < 0.05 and ** *p* < 0.01 vs. DSS group, ns means *p* > 0.05 vs. DSS group, *n* = 5. Table S1. The composition of diet for mice.
