# Supplementary material for: Protective Effect of the Polyphenol Ligustroside on Colitis Induced with Dextran Sulfate Sodium in Mice
Source: Nutrients. 2024 Feb 13;16(4):522. doi: 10.3390/nu16040522 (PMC10891938; doi:10.3390/nu16040522)
Supplement: Supplementary file 1 [file nutrients-16-00522-s001.zip › nutrients-2856302-supplementary.pdf]

Supplementary Materials

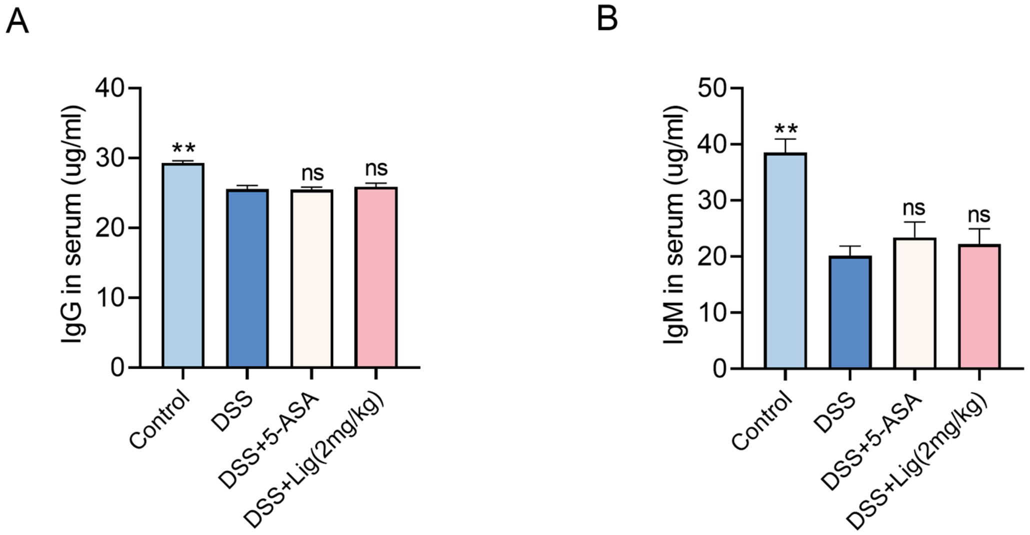

Supplementary Figure S1. The concentrations of IgG (**A**) and IgM (**B**) in serum. Data were presented as means ± SEM. * $p < 0.05$ and ** $p < 0.01$ vs. DSS group, ns means $p > 0.05$ vs. DSS group, $n = 5$.

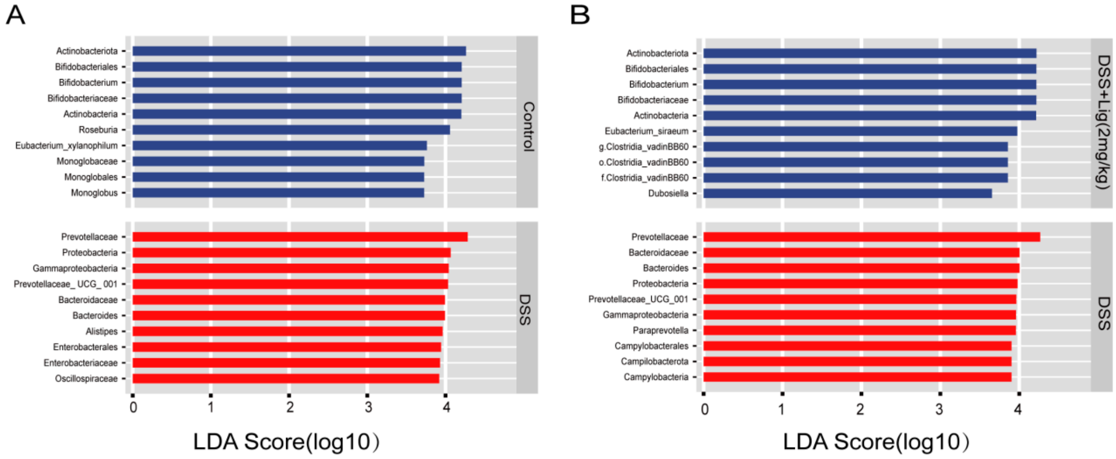

Supplementary Figure S2. Top ten Linear discriminant analysis (LDA) score for different taxa abundances (**A**) between control and DSS groups and (**B**) between DSS + Lig (2 mg/kg) and DSS groups, $n = 5$.

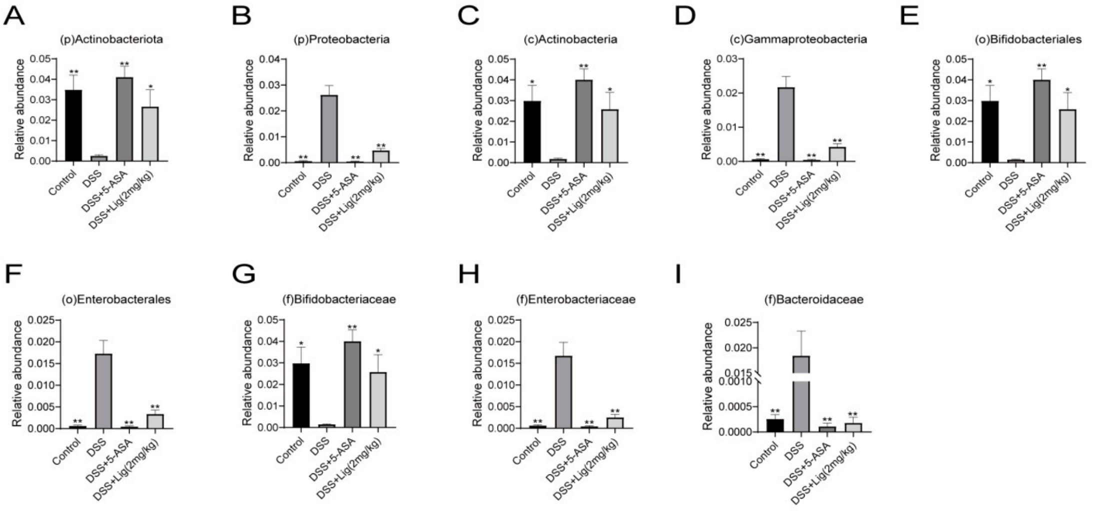

Supplementary Figure S3. Relative abundance of predominant bacteria is shown for the phylum (**A**,**B**), class (**C**,**D**), order (**E**,**F**) and family (**G**–**I**) levels. Data are presented as means ± SEM. * $p < 0.05$ and ** $p < 0.01$ vs. DSS group, ns means $p > 0.05$ vs. DSS group, $n = 5$.

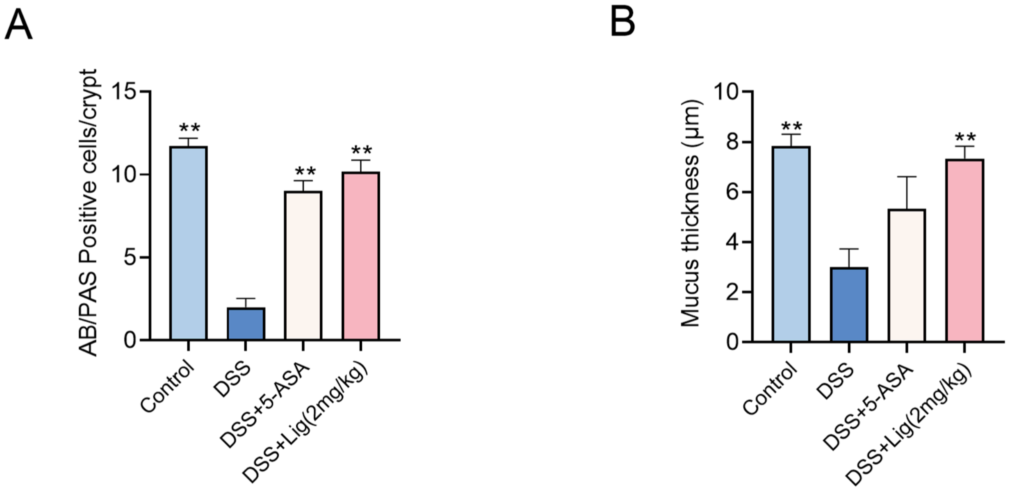

Supplementary Figure S4. (**A**) The quantification of AB/PAS Positive cells/crypt in colon tissue. (**B**) Mucus thickness of colon tissue. Data are presented as means ± SEM. * $p < 0.05$ and ** $p < 0.01$ vs. DSS group, ns means $p > 0.05$ vs. DSS group, $n = 5$.

Table S1. The composition of diet for mice.

**Energy Ratio**	
protein	20.6%
fat	12.0%
carbohydrate	67.4%
gross heat	3530 Kcal/kg
**Vitamin**	
Vitamin A, IU/kg	≥7000
Vitamin D, IU/kg	≥800
Vitamin E, IU/kg	≥60
Vitamin E, mg/kg	≥3.0
Vitamin B1, mg/kg	≥8
Vitamin B2, mg/kg	≥10
Vitamin B6, mg/kg	≥6
Vitamin B12, mg/kg	≥0.02
niacin, mg/kg	≥45
pantothenic acid, mg/kg	≥17
folacin, mg/kg	≥4.0
biotin, mg/kg	≥0.10
bilineurine, mg/kg	≥1250
Vitamin C, mg/kg	/
**Mineral Substance**	
Magnesium (Mg), g/kg	≥2.0
Potassium (K), g/kg	≥5.0
Sodium (Na), g/kg	≥2.0
Ferrum (Fe), mg/kg	≥100
Manganese (Mn), mg/kg	≥75
Copper (Cu), mg/kg	≥10
Zinc (Zn), mg/kg	≥30
Iodine (I), mg/kg	≥0.5
Selenium (Se), mg/kg	0.1~0.2
